# Obituary

**Published:** 1888-12

**Authors:** 


### Obituary.

DR. HENRY B. SANDS, of New York, died suddenly in his carriage while returning home from a visit to a patient, on Sunday P. M., November 18, 1888, in the fifty-ninth year of his age. Though his health had been somewhat impaired of late, he was yet in the active practice of his profession, and seemed in the best of spirits even up to within a few moments of his death, which was decided at the autopsy to have been due to heart failure.

Dr. Sands was concededly the leading surgeon of his native city, recognized as such not only by his dextrous skill as an operator, and his unerring acumen as a diagnostician, but by that other and even more important qualification, a keen and well-poised surgical judgment. These made him famous throughout the world, and led to his frequent summons as a consultant in difficult, dangerous, and distinguished cases. He will be remembered as having rendered conspicuous services in the management of the cases of General Grant, ex-Senator Conkling, and Dr. C. R. Agnew.

No attempt is made here to do justice to the memory of this remarkable man, and it is difficult to express in words the loss which the profession and community suffers in the sadly sudden death of Henry Berton Sands.

